# Inhibition of Muscle-Specific Protein Kinase (MuSK) Releases Organophosphate-Aged Acetylcholinesterase (AChE) from C2C12 Cells

**DOI:** 10.3390/toxics13100829

**Published:** 2025-09-29

**Authors:** Monica Moncada-Restrepo, Sarah Eysoldt, Jeronimo Medina, Valentina Di Guida, Jeremy W. Chambers

**Affiliations:** 1 Department of Biology & Microbiology, College of Natural Sciences, South Dakota State University, Brookings, SD 57007, USA; monica.moncadarestrepo@sdstate.edu; 2 Proscia, Inc., Philadelphia, PA 19103, USA; seysosarah@gmail.com; 3 Department of Environmental Health Sciences, Robert Stempel College of Public Health & Social Work, Florida International University, Miami, FL 33199, USA; 4 Department of Biological Sciences, College of Arts, Sciences, and Education, Florida International University, Miami, FL 33199, USA

**Keywords:** organophosphate, acetylcholinesterase, muscle-specific protein kinase (MuSK), countermeasure, chemical screening

## Abstract

Mechanistically, OPs inhibit acetylcholinesterase (AChE), an enzyme that terminates cholinergic transmission, triggering a sustained activation of acetylcholine receptors. A component of the treatment for OP intoxication is oximes as AChE reactivators. However, oximes may not be efficacious and could worsen OP effects. Further, dealkylation of the AChE-OP adducts prevents oxime reactivation. Therefore, other approaches are needed to rescue AChE activity. We propose that replacing aged extracellular AChE with active intracellular enzymes may be an effective approach. Thus, molecular screening was used to identify small molecules that could displace aged AChE. C2C12 myoblasts were treated with 20 μM of diisopropylfluorophosphate (DFP) for one hour, followed by a drug panel. AChE activity and surface abundance were measured after 6 h. From the chemical screen, a promising hit, Pz-1 (a tyrosine kinase inhibitor), was identified, which decreased surface AChE on DFP-exposed C2C12 myoblasts in a dose-dependent manner without impacting viability. Additionally, AChE presence and activity were recovered after washing and supplementing the media with 100 nM of acetylcholine. Biochemically, Pz-1 inhibits muscle-specific protein kinase (MuSK), a kinase that interacts with AChE. These results suggest that altering MuSK activity may disrupt protein–protein interactions, destabilizing AChE, which may lead to the discovery of new countermeasures for OP exposures.

## 1. Introduction

Organophosphates (OPs) are chemicals employed globally as recognizable pesticides and are more commonly identified as chemical weapons [1]. OPs potently inhibit acetylcholinesterase (AChE), increasing synaptic levels of the neurotransmitter acetylcholine (ACh), which culminates in the hyperstimulation of ACh receptors (AChRs) [2]. Acute exposures to OPs may present clinically as muscle contractions, respiratory suppression, seizures, and even death in high-dose exposures [1,2]. Despite the limited information regarding OP exposure and related death rates, the World Health Organization initially estimated that over 3,000,000 people are exposed to organophosphates each year, causing around 220,000 deaths. Of these, intentional and nonintentional poisoning from organophosphate-based insecticides accounts for 80% of the total exposures [3,4]. The true exposure and survival rates are likely underestimated due to underreporting and limited statistical data. The use of OP nerve agents against civilian populations includes recent attacks in Russia (2020), the United Kingdom (2018), Malaysia (2017), and the use of sarin in Syria [5,6,7]. The standard treatment for OP toxicity in the United States was developed in the late 1950s and comprises atropine, which inhibits the activities of muscarinic AChR, and oxime (i.e., 2-pralidoxime [2-PAM]) that reactivates a portion of AChE [8,9,10,11]. Unfortunately, recent meta-analyses have called into question the efficacy of oximes. For example, mortality and the need for ventilation support were not significantly improved for patients treated with atropine and 2-PAM compared to those treated with atropine alone. Additionally, these analyses showed a significant increase in the likelihood of developing intermediate syndrome in patients who received 2-PAM [12,13,14]. This syndrome appears 24 to 96 h after OP exposure and is marked by severe muscle weakness. Bennet et al. (2024) found that 2-PAM modestly reactivates AChE in rat skeletal muscle homogenates pretreated with sarin and VX surrogates (~51% and 42%, respectively), which could explain the low efficacy in treating nicotinic symptoms in muscle tissue [15]. This finding coincides with recent meta-analysis studies with exposed populations that illustrate that 2-PAM is either ineffective or may be adversely affecting health outcomes following OP exposures [12,16,17]. These studies highlight the critical need for new therapeutics capable of restoring active enzymes.

OPs inhibit AChE by adding a phosphyl group to the active site serine (Ser), rendering a phosphylated enzyme. Oximes are designed to adduct the phosphyl group and restore activities [1]. But many OP compounds, including sarin, diisopropyl fluorophosphate (DFP), and the insecticide parathion, can create irreversibly phosphylated forms of AChE, known as aged AChE, within minutes [18]. Aged AChE is immune to the actions of most oximes because the dealkylation of the initial phosphylated adduct fixes the phosphate in an extremely stable transition state-like conformation [18,19,20]. The aging process makes the phosphate group removal difficult, preventing reactivation by nucleophilic attackers like oximes [19]. There are efforts to generate more potent oxime species and cultivate realkylating compounds that would make aged AChE vulnerable to reactivation by traditional oximes [19,21]. These new compounds exhibit an inability to reactivate aged AChE by 2-PAM and poor blood–brain barrier permeability [19,20,22]. Additionally, these compounds have been rigorously examined against human erythrocyte AChE, but have yet to be thoroughly examined *in vivo* [20,22]. Thus, new approaches to recover AChE activity, of both phosphylated and aged enzyme, and restore proper cholinergic neurotransmission are needed, and surface enzyme turnover may represent an opportunity to fill this gap. 

AChE is a multi-protein complex on the outer leaflet of the post-synaptic membrane [23], where it interacts directly with the transmembrane tyrosine kinase (TK) muscle-specific protein kinase (MuSK) [24]. MuSK is recruited following the pre-synaptic release of Agrin, which interacts with LDL receptor-related protein 4 (Lrp4) [25]. The local MuSK activity triggers downstream of tyrosine kinase-7 (Dok7) dimer assembly via direct phosphorylation [24,26]. The Dok7 scaffolds facilitate signaling through adaptor proteins Crk and Crk-L that drive the assembly of AChR [25]. In the presence of active cholinergic signaling (i.e., elevated ACh), AChR engages the expression of AChR and AChE to handle elevated levels of neurotransmitter [27]. The MuSK complex is critical to AChE localization, as it is the foothold for AChE in the complex and at the synapse [24]. MuSK catalytic activity is necessary for the complex stability and disassembly [28,29,30]. Consequently, targeting members of the enzyme complex could allow the release of phosphylated enzymes, making way for new, active AChE from within the cell. 

The current study sought to identify small molecules that can release AChE from the surface of muscle cells without impacting cellular viability or the ability for new enzymes to migrate to the surface. A chemical screening of a drug panel that included Food and Drug Administration (FDA)-approved drugs was employed on mouse myoblast cells (C2C12) exposed to the organophosphate, diisopropylfluorophosphate (DFP), at a concentration and duration that aged 60% of the enzyme activity. One hit identified by the screen, Pz-1, was found to release aged AChE from the surface, and, after washing the cells, it did not impair recovery of AChE on the surface of myoblasts. Pz-1 was found to be an inhibitor of MuSK and catalytically competed with ATP for binding sites within myoblasts. These studies reveal that targeting the activities and interactions of MuSK may be a means to release inhibited enzymes, but it may also indicate that targeting intracellular members of the complex could be a route to restoring AChE following acute OP exposure. 

## 2. Materials and Methods

### 2.1. Materials

Recombinant MuSK (PV3834) was purchased from ThermoFisher (Waltham, MA, USA). Pz-1 ≥98% (HPLC) (N-(5-(Tert-Butyl)isoxazol-3-yl)-2-(4-(5-(1-methyl-1H-pyrazol-4-yl)-1H-benzo[d]imidazol-1-yl)phenyl)acetamide), SML1693, was acquired from Sigma Aldrich (St. Louis, MO, USA). PhosphoSens© substrate (AQT0704), ATP (AQT100XATP), DTT (AQT1000XDTT), EGTA (AQT1000XEGTA), enzyme dilution buffer (20 mM HEPES, pH 7.5, 0.01% Brij-35, 5% Glycerol, 1 mg/mL Bovine Serum Albumin, AQT1XEDG-noEGTA), and Reaction Buffer (500 mM HEPES, pH 7.5, 0.1% Brij-35, 100 mM of MgCl_2_) for the *in vitro* kinase assay were obtained from AssayQuant (Marlboro, MA, USA).

### 2.2. Cell Culture

Human IMR-90 lung fibroblasts (ATCC, CCL-186), human SH-SY5Y neuroblastoma cell lines (ATCC, CRL-2266), and C2C12 mouse myoblasts (ATCC, CRL-1772) were cultured according to the supplier’s recommendations (American Type Culture Collection, Manassas, VA, USA). SH-SY5Y cells were used as a positive control for AChE expression, and IMR-90 was to serve as an AChE-negative control. Briefly, cells were adapted to and cultured in Dulbecco’s Minimal Essential Media (DMEM) supplemented with 10% fetal bovine serum (FBS; Atlanta Biologicals, Flowery Branch, GA, USA), penicillin/streptomycin, and 5 µg/mL of plasmocin (both purchased from Fisher Scientific, Pittsburgh, PA, USA). The common media was used to prevent influence from different components in distinct media compositions on physiological outcomes. The cells were grown at 37 °C with 5% CO_2_ under constant humidity.

### 2.3. Cell Transfection 

For ectopic expression of AChE, plasmid DNA{Syed, 2008 #53}[pcDNA:AChE {Syed, 2008 #53} was a gift from Georg Weber (Addgene plasmid # 106442) (Addgene, Watertown, MA, USA)] was transfected separately into IMR-90 cells using the Fugene^®^HD (Promega, Madison, WI, USA) at a 3:1 ratio (reagent to DNA). pcDNA:AChE was diluted in serum-free, dye-free DMEM and combined with Fugene^®^ HD, and it was incubated at room temperature for 15 min before adding the mixture drop-wise to culture wells. After 8 h, fresh medium was added to the cells, and protein expression was monitored by On-Cell Western analysis. These cells served as the positive controls for the experiments.

### 2.4. On-Cell Western (OCW) for Surface AChE Detection 

On-cell westerns (OCWs) can be used to detect the relative abundance of proteins on the cell surface by fixing cells without permeabilization and detecting surface epitopes with antibodies [31,32]. Cultured cells were grown to ~80% confluency in black-walled, optically clear-bottomed 96-well plates, and the media was removed. The cells were washed twice in pre-warmed (37 °C) serum-free media and then gently fixed with 2% paraformaldehyde (PFA) for 10 min to secure the cells. The cells were washed again twice with serum-free media at room temperature. The cells were then incubated with 100 µL of LI-COR Blocking Buffer, after which a brief five-minute wash was performed with 1xTBS. Next, 100 µL of pre-warmed serum-free media with an AChE-specific antibody (Abcam, Cambridge, UK; ab31276) at 1 µg/mL was added to the wells, and the plate was incubated for 1-h at 37 °C, gently rocking. The wells were then washed three times for 5-min in 50 mL of prewarmed serum-free media. Next, the cells were incubated at 37 °C (gently rocking) for one hour with a mixture of LI-COR (Lincoln, NE, USA) CellTag^TM^ 700 stain and an IRDye© 800 anti-goat antibody (diluted 1:20,000) in serum-free media. The cells were then washed three times in 1xTBS and imaged using the LI-COR Odyssey CLX and Image Studio^TM^ Software (LICORBio, Lincoln, NE, USA) and analyzed using the LI-COR Empiria Studio software (version 3) (LICORBio, Lincoln, NE, USA). Statistical and quantitative analyses were performed with GraphPad Prism (version 9) (Boston, MA, USA).

Selectivity of the AChE Antibody used in the OCW assay was verified by conventional Western blot. Lysates from C2C12 and SH-SY5Y cell lines, which endogenously express AChE, as well as IMR-90 cells with transient AChE expression (pcDNA:AChE) were used as positive controls. Untransfected IMR-90 cells served as negative controls. Furthermore, binding specificity was validated using an AChE-blocking peptide and a scrambled, commercially available blocking peptide. 

### 2.5. Screening of Chemical Library 

C2C12 myoblasts were cultured to 80% confluency in a 96-well, black-walled, clear-bottom plate compatible with the AChE OCW approach. The OCW was optimized for screening with C2C12 myoblasts, AChE-expressing and untransfected IMR-90 cells. Reproducibility of the C2C12 myoblasts-based AChE detection was determined with a Z’ analysis. The cells were then treated with 20 µM of DFP for 1 h. The cells were then screened with compounds from the Library of Pharmacologically Active Compounds (LOPAC^1280^—Sigma-Aldrich, Burlington, MA, USA). For the primary screen, cells were either untreated, provided with 0.5 µL of DMSO, or the LOPAC compound to a final concentration of 20 µM in a 200 µL volume. The cells were incubated for 6 h to examine for acute cytotoxicity and potential robustness of effects. AChE OCW analyses were performed immediately after the incubation with compounds. Positive hits were those that reduced the level of AChE signal in the 800 channel of the OCW while exhibiting a minimal loss of CellTag^TM^ 700 signal (<15%). Compounds exhibiting significant cytotoxicity as evidenced by loss of CellTag^TM^ 700 intensity (>15%) were eliminated. Also, compounds that did not decrease AChE levels at the aforementioned concentrations were not considered hits. The screens were completed in triplicate. One hit was confirmed with dose-dependent increases in compound levels (0–100 µM) after DFP treatment.

### 2.6. AChE Activity Assay

An Ellman’s assay with each OP and cell lysate was performed to determine relative AChE activity. The assay comprised 0.5 mM of ACh in 40 mM phosphate buffer (pH 8.0) with 0.1 g/L Bovine Serum Albumin (BSA) and 1 mM 5,5′-dithio-bis(2-nitrobenzoic acid) (DTNB). The colorimetric change in the reaction mixture was measured at 410 nm at 25 °C in a BioTek Synergy H1 plate reader (Agilent, Santa Clara, CA, USA), and human erythrocyte AChE (Sigma—C0663, Burlington, MA, USA) was used to validate the assay. Four measurements (wells) were used for each replicate experiment.

### 2.7. Surface AChE Activity Assay

Ellman’s colorimetric technique was optimized to measure total and cell surface AChE activity in C2C12 myotubes [33,34]. In this assay, cellular AChE breaks down acetylthiocholine (ATCh) into acetate and thiocholine (TCh). The thiol moiety in TCh subsequently reacts with the non-cell-permeable 5,5′-dithiobis-(2-nitrobenzoic acid) (DTNB) to yield 5-Thio-2-nitrobenzoate (TNB^2−^), a yellow compound measurable at 412 nm. First, the C2C12 myoblasts were plated in 96-well plates previously coated with poly-L-ornithine and laminin, at a density of 2500 cells per well. The growth medium used was DMEM, enriched with 2 g/L of NaHCO_3_, 20% heat-inactivated fetal bovine serum (FBS), 100 units/mL of penicillin, and 100 μg/mL of streptomycin. Differentiation into myotubes was induced two days later by replacing the growth medium with a differentiation medium containing DMEM, 2 g/L of NaHCO_3_, 5% heat-inactivated horse serum (HS), 100 units/mL of penicillin, and 100 μg/mL of streptomycin; this day was designated as “Differentiation Day 0”. C2C12 cells fully differentiate into myotubes after six days of incubation with differentiation medium. Since AChE levels increase during differentiation and stabilize once the cells are fully differentiated [35], for the following five days, 60% of the differentiation medium was replaced daily. The C2C12 myotubes were then used for the AChE activity assay on “Differentiation Day 6.”

For assessing surface activity in live C2C12 myotubes, cells were first rinsed with Hanks’ Balanced Salt Solution (HBSS) containing 0.4926 mM of CaCl_2_ and 0.9009 mM of MgCl_2_ (HBSS-CM). Subsequently, 100 μL of HBSS-CM was added to each well, followed by 50 μL of 2 mM DTNB and 50 μL of 0.04–8 mM ACh. TNB^2−^ formation was followed by measuring absorbance at 412 nm every minute for 30 min using a microplate reader (BioTek Synergy H1 hybrid reader). To avoid cell detachment, DTNB and ATCh solutions were prepared in HBSS-CM. To obtain the total activity, the myotubes were lysed by incubating with 50 μL of HBSS-CM containing 0.5% Triton X-100 for 15 min after the rinsing step. To keep the final volume equal to surface activity, 50 μL of HBSS-CM was added before adding 50 μL of 2 mM DTNB and 50 μL of the ACh solutions. A control containing only DTNB and myotubes was included to account for any non-specific reaction of the reagent with other thiols on the cell surface. Both assays were done at room temperature. The kinetic plots of absorbance at 412 nm against time were used to determine the slope, which allowed the calculation of the initial velocity (V_0_) expressed in μM TNB^2−^/min via the Beer–Lambert law (Equation (1)).

(1) $$V_{0}\left(\mu M\;TNB^{2-}/min\right)=\frac{{\Delta A}_{412nm}/min}{L\left(cm\right)*\;\varepsilon (M^{-1}{cm}^{-1})}*1000(\mu M\;M^{-1}),$$

In this equation, ΔA_412_ nm/min denotes the slope, and L is 0.56 cm, representing the light path length through 96-well plates. The extinction coefficient for TNB^2−^ at 412 nm is ε = 14,150 M^−1^ cm^−1^, and 1000 (μM M^−1^) serves as the conversion factor from M to μM. V_0_ values at different ATCh concentrations were used to obtain Michaelis–Menten plots and calculate surface and total AChE kinetic parameters. Km and Vmax were estimated by a nonlinear regression of the Michaelis–Menten plot, using GraphPad Prism 10 software (Boston, MA, USA).

### 2.8. Western Blotting Analyses

Cells were lysed with Radioimmunoprecipitation assay buffer (RIPA, Millipore Sigma, Burlington, MA, USA) supplemented with Protease inhibitors (1 mM of PMSF and cOmplete™ ULTRA Protease Inhibitor Cocktail (Roche, Basel, Switzerland) and phosphatase inhibitors (phosSTOP phosphatase inhibitor cocktail (Roche)). Lysates were incubated on ice for 30 min, followed by sonication and centrifugation at 15,000× *g* and 4 °C for 15 min. The protein concentration of the supernatants was measured using the bicinchoninic acid (BCA) assay (Thermo Scientific, Waltham, MA, USA, Cat. 23225) following the manufacturer’s instructions. Equal amounts of samples were prepared in Laemmli buffer containing 10% (*v*/*v*) β-mercaptoethanol. Samples were resolved by sodium dodecyl sulfate polyacrylamide gel electrophoresis (SDS-PAGE) and transferred to Polyvinylidene fluoride membranes (PVDF, Immobilon™—Millipore, Bedford, MA, USA) or nitrocellulose membranes using a Bio-Rad TurboTransfer system. Membranes were blocked at room temperature for 1 h prior to overnight incubation at 4 °C with primary antibodies prepared in blocking solution (Supplementary Table S1). The membranes were washed three times for seven minutes with Tris-Buffered Saline (TBS) containing 0.1% (*v*/*v*) Tween 20 (0.1% TBST) and incubated for 1 h at room temperature with fluorophore-conjugated secondary antibodies Anti-rabbit IgG DyLight 800 4X PEG Conjugate (#5151S), Anti-mouse IgG DyLight 680 Conjugate (#5470S) from Cell Signaling Technology, Inc. (Danvers, Massachusetts, USA), and IRDye^®^ 800CW anti-Goat IgG (# P/N 096-68074) from Li-COR Biosciences (Lincoln, NE, USA). After the secondary antibody incubation, the membranes were washed three times with 0.1% TBSt for seven minutes at room temperature. Fluorescent signals at 700 and 800 nm were detected using the Odyssey CLx Imaging System (Li-COR Biosciences, Lincoln, NE, USA). Fluorescent bands of the protein of interest and a housekeeping protein were quantified with Empiria Studio Software, version 3 (Li-COR Biosciences, Lincoln, NE, USA). The relative protein expression levels were determined by normalizing the fluorescence of the proteins of interest against the housekeeping protein (LI-COR Biosciences, Lincoln, NE, USA).

### 2.9. In Vitro MuSK Kinase Assay 

The in vitro phosphorylation activity of the catalytic domain of the recombinant human MuSK was measured using the PhosphoSens© assay based on the Chelating enhanced effect or ChEF principle (AssayQuant, Marlboro, MA, USA). The assay uses a peptide bearing an 8-hydroxyquinoline derivative (sulfonamide-oxine, Sox) moiety and an adjacent and phosphorylatable tyrosine, serine, or threonine residue. The peptide coordinates to Mg^2+^ with low affinity when unphosphorylated and with high affinity upon phosphorylation due to the additional chelating effect of the phosphate group, enabling the detection of phosphorylation events through the chelate fluorescence. MuSK activity was measured using the PhosphoSens© peptide AQT0704 (AssayQuant, Marlboro, MA, USA). as substrate, and the kinase inhibition in the presence of Pz-1 was obtained. To start the assay, 100× Pz-1 stock solutions were prepared in DMSO and diluted to 10× in a HEPES buffer (19.4 nM HEPES, pH 7.5, 4.9% glycerol, 0.0092% Brij-35, 0.97 mg/mL of BSA, 0.1 mM of EGTA, 1 mM of DTT, 10% DMSO). The MuSK catalytic subunit (250 nM of MuSK, in 3.8 mM Tris, pH 7.5, 11.3 mM of NaCl, 0.038 mM of EDTA, 0.0016% Triton X-100, 1.1 mM of DTT, 19.6 mM HEPES, pH 7.5, 0.092 mM of EGTA, 0.085% Brij-35, 0.85 mg/mL of BSA, 8.3% glycerol,) was then mixed with varying concentrations of 10× Pz-1 at a 2:1 ratio and preincubated with gentle shaking for 1 h at 30 °C. Simultaneously, a reaction mixture containing 14.3 μM of PhosphoSens© peptide AQT0704, 1.43 μM of ATP, 1.43 mM of DTT, 0.79 mM of EGTA, 71.43 mM HEPES, pH 7.5, 0.014% Brij-35, and 14.23 mM of MgCl_2_ was prepared and equilibrated for 5 min before the reaction started at 30 °C. Subsequently, 3 μL of each MuSK-Pz-1 solution was added to a prewarmed 384-well plate (Low-Volume, White Round-Bottom Polystyrene, Corning, Corning, NY, USA), followed by 7 μL of the reaction mixture (final concentrations: 55.5 mM HEPES, 0.76 mM of Tris, 2.3 mM of NaCl, 0.0076 mM of EDTA, 0.00032% Triton X-100, 1.1 mM of DTT, 0.58 mM of EGTA, 0.028% Brij-35, 0.27 mg/mL of BSA, 2.2% Glycerol, 50 nM of MuSK, 10 μM of PhosphoSens© peptide AQT0704, 1.0 μM of ATP, and 10 mM of MgCl_2_, 1% DMSO and 0–100 nM of Pz-1). A blank, excluding the enzyme and the inhibitor, was utilized to subtract background fluorescence. The plate was sealed, and fluorescence measurements were performed every 3 min for 240 min at 30 °C using a BioTek Synergy H1 Hybrid Plate Reader with excitation/emission wavelengths of 360/492 nm. The initial velocities (RFU/min) were calculated by plotting RFUs against time and obtaining the slope of the linear range of the curve using GraphPad Prism (La Jolla, CA, USA). Pz-1 IC_50_ was then determined via a four-parametric logistic curve of the Velocity versus the Log concentration of Pz-1.

### 2.10. Statistical Analysis

Biochemical and other cellular measures were done with a minimum of three experimental replicates. IC_50_ values and statistical differences were calculated by using GraphPad Prism version 10.0 (GraphPad Software, San Diego, CA, USA) with nonlinear regressions and analyses of variation, as indicated below. IC_50_ values for Pz-1 were calculated using the inhibitor versus normalized response curve fitting function (variable slope). To determine statistical significance, an unpaired *t*-test analysis was employed for significance between two groups, and one-way ANOVA for more 3 or more group treatments. Statistical significance is indicated by an asterisk in figures in which the *p*-value is less than 0.05. Data are displayed as means with error bars representing plus and minus one standard deviation.

## 3. Results

### 3.1. Surface AChE Contributes to Significantly Less Total AChE Activity than Intracellular Pools in C2C12 Cells

**Figure 1:**
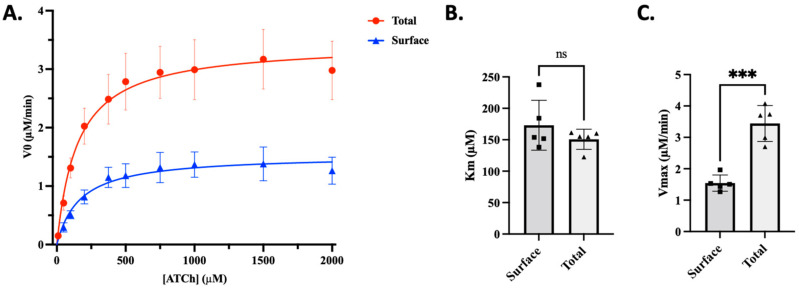
Intracellular AChE activity represents the majority of cellular AChE enzymatic activity. A modified Ellman’s assay with no detergent was developed to distinguish surface AChE activity from total enzyme activity in differentiated C2C12 myotubes. (**A**) Michaelis–Menten plot of cell surface and total AChE activity. (**B**) Km comparison between surface and Total AChE activity. (**C**) Vmax comparison between surface and Total AChE activity. Using the measurements of total AChE and surface AChE activities, one can derive intracellular enzymatic activity levels, which reveal that ~60% of the enzymatic activity is contained within the cell. The asterisks (***) demonstrate a statistical difference (*p* < 0.001) established by an Unpaired *t*-test; ns = no significant. Data are shown as the mean ± SD of 5 independent experiments.

OPs inhibit AChE in both the central nervous system and the periphery. A major contributor to peripheral OP symptoms, particularly muscular weakness and respiratory distress, is the inhibition of AChE in skeletal muscles [36]. To emulate one of the target tissues of OPs, C2C12 cells derived from mouse skeletal muscle myoblasts were used. This immortalized cell line is a subclone [37] of mouse myoblast cells established by Yaffe and Saxel in 1977, which can differentiate into myotubes [38]. They allow reproducibility and uniformity during the early stages of high-throughput drug screening assays in muscle cells. In addition, these cells are AChE-positive, allowing for the study of the dynamics of surface AChE in skeletal muscle cells. First, to test the hypothesis that inducing a decrease in aged AChE on the cell surface creates space for native enzymes, it was necessary to discern the abundance of AChE activity between the cell surface and intracellular compartments. For this, an assay that could distinguish between surface and intracellular AChE activity was developed. Using this approach, the enzymatic activity in differentiated C2C12 myotube lysates (total) and surface activities on myotubes with intact membranes were measured. According to the kinetic plots in Figure 1A, the enzyme activities displayed very similar K_m_ values (Figure 1B); however, there was a ~2.5-fold difference in the Vmax (Figure 1C), revealing that more enzymatic activity could be attributed to non-surface (intracellular) enzyme pools.

### 3.2. AChE On-Cell Western Assay Identifies Chemicals Capable of Reducing Surface AChE Levels in C2C12 Myoblast-like Cells

**Figure 2:**
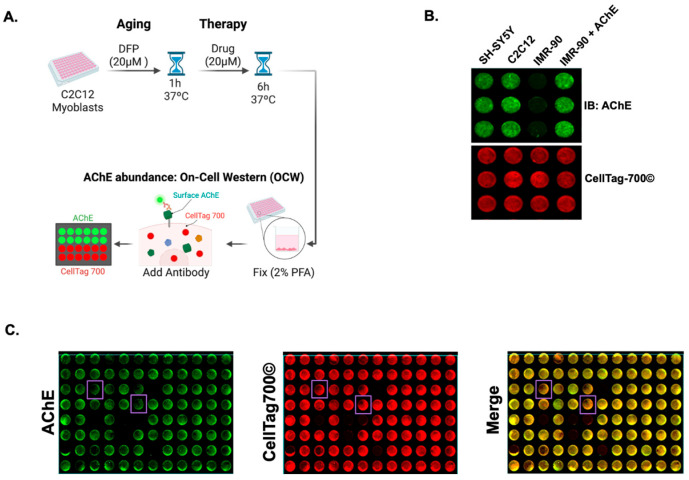
An OCW-based screen to assess surface protein levels for AChE identifies chemicals capable of decreasing surface AChE. (**A**) Schematic representation of the AChE OCW. Cells were treated with 20 µM of DFP for 1 h to allow time for aging. Following that time, cells were treated with 20 µM of individual compounds from the LOPAC^1280^ library. Cells were then gently fixed and assessed for the relative surface abundance with an anti-AChE antibody, with cell viability monitored by the CellTag© 700 dye. Lastly, the plates were measured using a LiCOR Odyssey CLX scanner. Created in BioRender. Moncada-Restrepo, M. (2025) https://BioRender.com/iulbiu7, accessed on 31 July 2025. (**B**) Cells with previously known abundances of AChE were used to determine the accuracy and establish the basal parameters of the OCW high-throughput assays. Human neuroblastoma (SH-SY5Y cells) and mouse myoblast-like (C2C12) cells were known to express AChE, while human IMR-90 lung fibroblasts did not have detectable AChE. Ectopic expression of AChE in IMR-90 cells was detected by the OCW. Cell viability/abundance was determined with CellTag© 700. (**C**) A representative plate using the AChE OCW on C2C12 pre-treated with DFP, followed by incubation with drugs of the LOPAC^1280^ library, where each well represents a unique compound. The green signal demonstrates the relative abundance of AChE following drug treatment, while the red signal indicates cellular viability. Hits were defined as signals that retained their CellTag© 700 signal (>85%—unaltered cell viability) while demonstrating a loss of >50% of the control AChE signal (decreased green fluorescence); examples of pz-1 signal changes are highlighted with lavender boxes.

**Figure 3:**
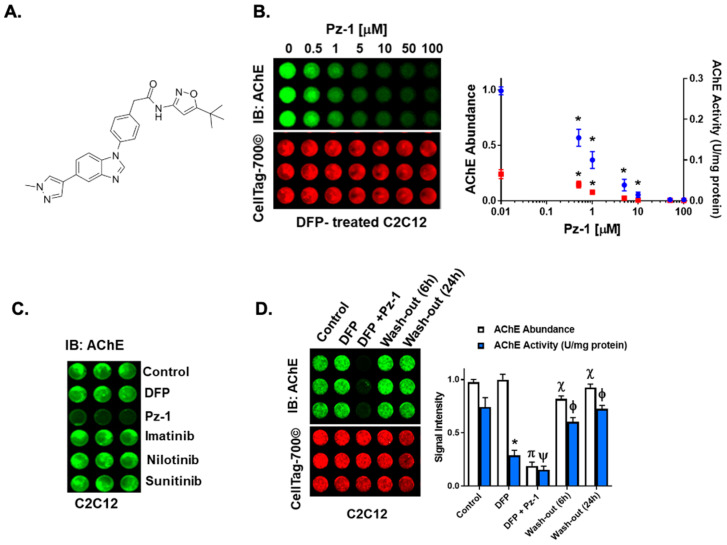
Pz-1, an inhibitor of AChE-interacting MuSK, destabilizes surface AChE, leading to enzyme turnover. (**A**) Chemical structure of Pz-1. (**B**) Pz-1 validation of surface AChE destabilization was performed using the AChE OCW in a dose-dependent manner. Undifferentiated C2C12 myoblasts were subjected to 20 µM of DFP for 60 min and followed by increasing concentrations of Pz-1. AChE levels and acute cell viability were assessed by OCW (left panel). The relative abundance of surface AChE determined by OCW (blue dots) was compared to total C2C12 myoblast AChE activity (red squares) using an Ellman’s assay. The * indicates significant differences in cells treated with DFP and Pz-1 when compared to cells treated with DFP only (control). Differences between the treatments and control were calculated with nonlinear regressions using the Dose Shift analysis in GraphPad Prism. (**C**) As Pz-1 is a RET-tyrosine kinase family inhibitor, additional tyrosine kinase inhibitors (20 µM/60 min) were examined by OCW for AChE. (**D**) C2C12 myoblasts were subjected to 20 µM of DFP in the presence and absence of 5 µM Pz-1. Cells were washed, and their medium was replaced with normal culture media for 6 or 24 h. AChE and cellular viability were assessed in OCW. Quantified values for AChE relative abundance and total cellular activity were assessed. The * indicates a difference between Control and DFP-treated cells, while π indicates significance between DFP-treated abundance and DFP/Pz-1-treated abundance. The Ψ designates a difference between DFP-treated activity and DFP/Pz-1-treated activity. Lastly, χ and Φ indicate differences between wash-out conditions and DFP/Pz-1-treated conditions according to one-way ANOVA.

Next, an OCW to measure AChE surface abundance on C2C12 myoblast-like cells was developed (outlined in Figure 2A). Human SH-SY5Y neuroblastoma cells, mouse C2C12 myoblast-like cells, and IMR-90 cells were fixed and stained against AChE. It was found that surface levels of AChE were detected on the enzyme-positive SH-SY5Y cells and C2C12 cells (Figure 2B) but not on IMR-90 cells, which have negligible AChE levels. The selectivity of AChE antibody was verified by transfecting IMR-90 cells with recombinant AChE and detecting surface enzyme (Figure 2B). The reliability of the OCW assay was also evaluated, obtaining a Z’ score of 0.78, with C2C12 cells as positive controls and IMR-90 cells as negative controls. (Figure 2B). Next, the OCW assay was used to identify small molecules capable of inducing the removal of aged AChE levels from the plasma membrane of C2C12 myoblast-like cells. To this end, the C2C12 cells were pre-treated with 20 µM of the organophosphate DFP for 1 h. This dosage resulted in approximately 60% AChE aging without impacting cell viability. Then, the cells were treated with 20 µM of a small battery of the LOPAC^1280^ library for 6 h. We looked for a decrease in AChE surface abundance, represented by a decreased green fluorescence, with no changes in cell density, indicated by unaltered red fluorescence, as shown by the lavender boxes (Figure 2C). Hits were characterized as those wells that demonstrated marked decreases in AChE surface levels of >50% of the control signal and retained their cellular viability (indicated by CellTag700© signal) with >85% of signal retention. Out of the tested compounds, 40 were found to decrease aged AChE surface levels without affecting cell density (Figure 2C). Among the hits, Pz-1, a benzimidazole-based tyrosine kinase inhibitor, emerged as a potent candidate for AChE surface removal (Figure 3A) because of its superior impact on surface AChE levels compared to other hits. Pz-1 caused an ~82% decrease in AChE signal in myoblasts without impacting viability (94% of control viability signal). This inhibitor exhibits selectivity for the Rearranged during transfection (RET) receptor, the Vascular endothelial growth factor receptor 2 (VEGFR2) (IC_50_ < 1 nM), and the AChE-interacting protein Muscle-Specific Tyrosine Kinase (MuSK) receptor (K_d_ < 50 nM) [39].

### 3.3. Pz-1 Decreases AChE Surface Levels in a Dose-Dependent Manner

To characterize the effect of different concentrations of Pz-1 on surface AChE levels, AChE aging in C2C12 myoblasts was induced with 20 µM of DFP for 1 h, followed by a 6 h treatment with 0 to 100 µM Pz-1. Figure 3B shows a significant decrease in surface enzyme as the concentration of Pz-1 increased. Concentration at and above 0.1 µM led to a reduction of over 50% in AChE surface levels, with 50 µM and 100 µM resulting in barely detectable levels of the protein.

To ascertain the impact of the decline in esterase surface abundance on the active cellular pool of AChE following DFP treatment, Ellman’s assay was used to measure AChE activity in lysed cells treated under the same conditions. As expected, DFP alone decreased total AChE activity by more than 50%. Additionally, the presence of Pz-1 caused a further reduction in enzyme activity starting with the lowest tested concentration of Pz-1, while total activity abrogation was observed above 10 µM of Pz-1 (Figure 3B). With these curves, we were able to determine the EC_50_ of pz-1 for surface AChE levels to be 0.87 µM (±0.39 µM), and the IC50 for the remaining AChE activity was 1.05 µM (±0.42 µM), suggesting the loss of enzyme level and activity coincided.

Since Pz-1 targets more than one tyrosine kinase (TKs), it was unclear if its effect on surface AChE was specific to MuSK or resulted from a broader TK inhibition. To investigate this, the impact of other TK inhibitors on AChE levels at the cell surface was examined, including those targeting RET and VEGFR but not MuSK at the tested concentrations. (Figure 3C). Interestingly, only Pz-1 showed an ability to reduce AChE levels.

### 3.4. Exchanging Culture Media Restores AChE in DFP-Exposed Myoblasts Treated with Pz-1

To determine if AChE could be restored on the cell surface following its Pz-1-induced decline, the culture medium from C2C12 cells with DFP-aged AChE treated with 5 μM of Pz-1 was removed. The cells were refed with serum-free media containing 100 nM of ACh for 6 and 24 h to induce sustained cholinergic transmission and AChE expression. As shown in Figure 3D, AChE levels were substantially recovered, almost reaching the untreated levels. Remarkably, despite DFP causing a drastic reduction in enzymatic activity and the further decrease induced by Pz-1, the enzyme function was almost completely regained after the incubation times had passed.

**Figure 4:**
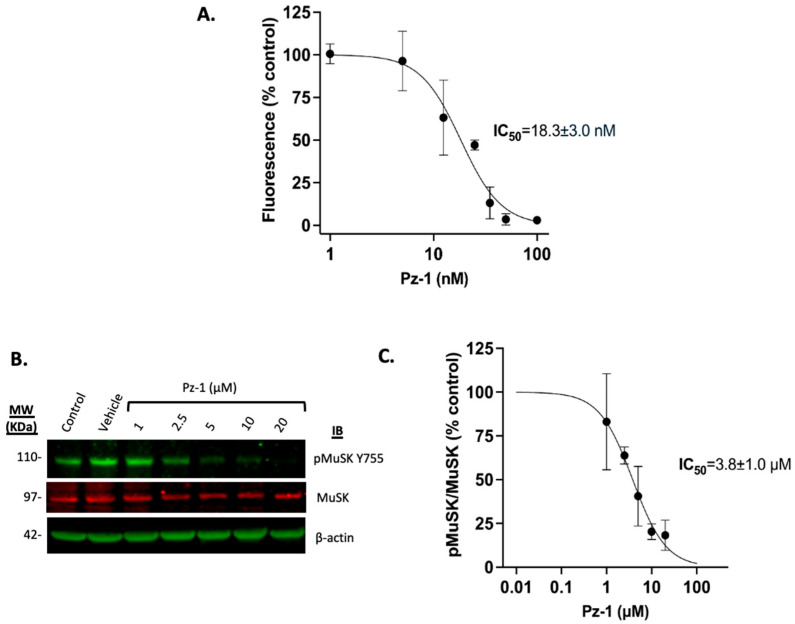
Pz-1 inhibits MuSK in C2C12 myotubes and in vitro. (**A**) Biochemical inhibition of MuSK using Pz-1 was assessed using an in vitro assay to calculate the Pz-1 IC_50_ on recombinant MuSK catalytic domain. (**B**) Western blot analyses were performed to monitor MuSK activation via phosphorylation (Y755); phospho-MuSK levels were normalized to total MuSK with actin serving as an additional control. (**C**) Analysis of Pz-1 inhibition using a dose–response curve plot to calculate the cell-based IC_50_. Data are shown as the mean ± SD of at least 3 independent experiments.

Previous work by Frett et al. 2015 demonstrated that Pz-1 interacts with MuSK (K_d_ < 50 nM) [39]. To verify that MuSK is a target of Pz-1, its impact was assessed on the tyrosine phosphorylating activity of MuSK in vitro, using the fluorescence-based PhosphoSens^®^ Kinase assay (AssayQuant Tech, Marlboro, MA, USA). Preincubation with increasing concentrations of Pz-1 for 1 h, followed by incubation with PhosphoSens© peptide, demonstrated that Pz-1 effectively impaired MuSK tyrosine kinase activity in vitro within the nanomolar range. More than a 50% decrease in fluorescence signal was observed at Pz-1 concentrations at and above 25 nM (IC_50_ = 18.3 ± 3.0 nM), and a complete inhibitory effect was achieved at 50 and 100 nM (Figure 4A).

Subsequently, to confirm MuSK as a target of Pz-1 in C2C12 cells, its inhibitory effect on C2C12 myotubes was evaluated by treating them with various concentrations of Pz-1 (1 to 20 µM) for 1 h (Figure 4B,C, Figure S1) and measuring the phosphorylation state of MuSK at Y755, a residue that becomes phosphorylated upon activation of the receptor. MuSK phosphorylation levels at Y755 show that Pz-1 inhibits MuSK in a dose-dependent manner in C2C12 myotubes, with an IC_50_ of 3.8 ± 1.0 µM. 

## 4. Discussion

The robustness of AChE activity is orchestrated through a variety of mechanisms that include signal transduction and tissue-specific protein–protein interactions to establish the proper regulation of neurotransmission [40]. Consequently, components within these regulatory pathways may possess untapped potential for countering OP exposures. In the current study, it was evaluated whether the disruption of the activities of these pathways and proteins could evoke turnover of OP-inhibited AChE, resulting in the removal and replacement of impaired enzyme, and culminating in the restoration of AChE activity. For this, an on-cell western (OCW) assay (Figure 2) for AChE was developed to screen against a chemical library to exploit cellular activities regulating surface AChE levels. The drug screen identified Pz-1, an inhibitor of tyrosine kinases, including MuSK [39], as a molecule capable of reducing OP-inhibited AChE levels, paving the way for new enzymes to reach the myoblast surface following removal of Pz-1. This study identifies that proteins and pathways regulating the expression, transport, and surface localization of AChE could be promising targets against current and emerging OPs.

Because AChE is stably anchored in the extracellular space, with newly synthesized protein being inserted at a very slow rate [40], we attempted to transiently modulate the localization and turnover of AChE as a strategy to replace aged AChE with nascent intracellular AChE. Considering that, during sustained cholinergic transmission, as the one induced by AChE inhibitors like DFP, AChE synthesis increases, but only a small portion of the secreted enzyme is retained on the cell surface [40], we surmised that inducing the disengagement of aged AChE from the plasma membrane would make way for native enzymes. The identification of Pz-1 demonstrated the feasibility of removing the aged surface enzyme (Figure 3); however, removal of Pz-1 by washout followed by 6-h incubation was done to restore AChE surface levels. OPs and Pz-1 have both relatively short biological half-lives [39,41]. Thus, the wash-out step of these drugs helps mimic their rapid in vivo metabolism. Due to the irreversible nature of the AChE-OP adduct and the slow turnover of the enzyme [40], even when the OPs have been cleared out, aged AChE will remain present, which signifies a continued disruption of the acetylcholine-dependent signaling, at least until the aged enzyme has been replaced with an active one. These results would first imply that transiently acting agents could be employed to target these AChE turnover pathways following OP exposures.

**Figure 5:**
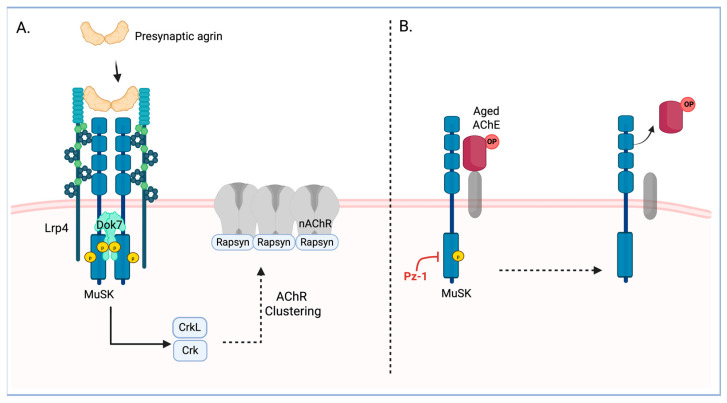
MuSK basal activity is involved in modulating surface AChE in C2C12 cells. (**A**) MuSK canonical pathway is induced by the binding of presynaptic agrin to the coreceptor LrP4, as well as the subsequent intracellular trans-autophosphorylation and full activation of the receptor. The activated receptor phosphorylated the adapter protein Dok7, which modulates the formation of clusters of the nicotinic Acetylcholine receptors (nAChR). (**B**) In muscle cells, extracellular AChE directly interacts with the MuSK extracellular domain. Our results suggest that inhibition of basal MuSK activity by Pz-1 induces the removal of aged AChE from the cell membrane of C2C12 cells. Created in BioRender. Moncada-Restrepo, M. (2025) https://BioRender.com/q380i59, accessed on 31 July 2025.

While it was demonstrated that Pz-1 can inhibit MuSK (IC_50_ ~ 18 nM; Figure 4), Pz-1 potent inhibition of RET, VEGFR2, as well as MuSK [39], led us to interrogate whether other tyrosine kinase inhibitors were able to decrease surface AChE. Figure 3C demonstrated that a small panel of alternative tyrosine kinase inhibitors was unable to replicate the effects of Pz-1. The observation that AChE turnover occurred only with Pz-1, and not with any other tyrosine kinase inhibitors [42,43], suggested that AChE modulation was selective to MuSK catalytic activity. This interpretation is supported by the confirmation that MuSK is a biochemical and cellular (IC_50_ ~ 3.8 mM) target of Pz-1. The association of the catalytic activity of MuSK with surface AChE levels implies that the change exerted by Pz-1 on MuSK may be exerted conformationally through protein–protein interactions within the MuSK-AChE complex or by yet-to-be-identified signaling pathways related to MuSK activity. While this study primarily investigated Pz-1’s capacity to modulate surface AChE and replace surface-aged AChE through MuSK, future studies will aim to investigate the effect of Pz-1 on key proteins of MuSK-related signaling pathways, such as Dok7 or LrP4 (Figure 5), and their impact on surface AChE.

Targeting proteins with the MuSK-AChE complex nexus (Summarized in Figure 5) may have potential risks with long-acting (poorly reversible or slow elimination) compounds or recurrent use. Loss of AChE complex proteins (or their activities) may lead to the dissolution of the complex and AChR receptor, as in myasthenia syndrome and myasthenia gravis [23,30]. Mutations in AChE complex genes or autoimmune targeting of AChE complex members result in the loss of AChE at neuromuscular synapses, diminishing synaptic integrity [23,44,45]. Thus, therapies targeting aged AChE turnover should be short-term, with short half-lives, to sustain active neuromuscular cholinergic synapses. In this sense, a readily available source of intracellular enzyme is critical to achieve a moderately fast surface AChE turnover (as illustrated with pz-1 in Figure 5B). In this regard, consistent with previous findings in chick muscles, the results on surface and total AChE activity showed that the availability of an active intracellular enzyme pool is more abundant than the surface-bound enzyme [46].

A caveat to this mechanism may be that most OP species are highly lipophilic and can gain access to intracellular compartments. Because ~2.5-fold more enzyme activity resides within muscle cells (Figure 1), it is likely that substantial pools of intracellular AChE may be inhibited, which might delay the actions of Pz-1, or require several rounds of Pz-1 administration and washout post-exposure to restore normal levels of surface AChE activity. The current study shows modest impacts using a myoblast model with Pz-1 washout, suggesting that minor inhibition of intracellular AChE was present in this system. This could significantly differ in myotubes, muscle explants, and in vivo studies. Therefore, a careful examination of both surface and total protein levels and activity is needed to ensure that active AChE is present in sufficient quantities following treatment. To address this, we developed an Ellman-style assay that could distinguish between surface and total cellular AChE activity (Figure 1). This approach of monitoring both surface levels and activity can allow for the relative determination of the percentage of active surface AChE.

While our findings show promising results using a C2C12 cell-based model, there are limitations to this study. The simplified nature of the cell-based model does not account for the complexity of physiological conditions. For instance, the lipophilic nature of most OPs can lead to their accumulation in adipose tissue in vivo, particularly in severe cases of intoxication [47,48]. This may result in a slow and prolonged release of the OPs, potentially sustaining AChE inhibition over time and hindering the pool of active and available enzyme that could replace the aged AChE. In addition, the relatively rapid metabolism of Pz-1 observed in its pharmacokinetic studies [39] may indicate the need for repeated administration of the inhibitor in patients with sustained AChE inhibition. Future in vivo studies that include monitoring OP levels in serum and urine, along with serum AChE activity, and determining the time frame of surface AChE recovery in muscle tissue would be crucial to better correlate these parameters with the in vivo efficacy of this approach.

Our results suggest for the first time that the catalytic activity of MuSK, a protein that directly interacts with AChE, may modulate the stability and/or turnover of AChE. This suggests that targeting this modulation could be a viable strategy for restoring cholinergic transmission after OP exposure. Future research should aim to explore the molecular pathways of MuSK involved in AChE modulation and identify more effective and selective MuSK inhibitors. Collectively, we assert that the continued interrogation of pathways regulating the expression, anchoring, and turnover will be essential to countering the effects of current and emerging OPs.

## Data Availability

The original contributions presented in this study are included in the article. Further inquiries can be directed to the corresponding author.

## Supplementary Materials

The following supporting information can be downloaded at: https://www.mdpi.com/article/10.3390/toxics13100829/s1, Table S1. List of antibodies used for Western Blot. Figure S1. Full Western Blot images for Figure 4B.

## Abbreviations

The following abbreviations are used in this manuscript:

2-PAM	Pralidoxime
ACh	Acetylcholine
AChE	Acetylcholinesterase
AChR	Acetylcholine Receptor
ATCC	American Type Culture Collection
ATCh	Acetylthiocholine
BSA	Bovine Serum Albumin
DFP	Diisopropyl Fluorophosphate
DM	Differentiation Medium
DMEM	Dulbecco’s Modified Eagle Medium
DMSO	Dimethyl Sulfoxide
DTNB	5,5′-Dithiobis(2-nitrobenzoic acid)
EDTA	Ethylenediaminetetraacetic Acid
FBS	Fetal Bovine Serum
FDA	Food and Drug Administration
HBSS	Hanks’ Balanced Salt Solution
HBSS-CM	HBSS containing 0.4926 mM of CaCl2 and 0.9009 mM of MgCl_2_
HS	Horse Serum
LrP4	Low-Density Lipoprotein Receptor-Related Protein 4
MuSK	Muscle-Specific Protein Kinase
OP	Organophosphate
OCW	On-Cell Western
RET	Rearranged during transfection
TBS	Tris-Buffered Saline
TCh	Thiocholine
TNB^2−^	5-Thio-2-nitrobenzoate
V_0_	Initial Reaction Velocity
VEGFR2	Vascular endothelial growth factor receptor 2
Vmax	Maximum Reaction Velocity
