# Supplementary material for: Inhibition of Muscle-Specific Protein Kinase (MuSK) Releases Organophosphate-Aged Acetylcholinesterase (AChE) from C2C12 Cells
Source: Toxics. 2025 Sep 29;13(10):829. doi: 10.3390/toxics13100829 (PMC12567851; doi:10.3390/toxics13100829)
Supplement: Supplementary file 1 [file toxics-13-00829-s001.zip › toxics-3750385-supplementary.pdf]

Supplementary material for

Manuscript title: Inhibition of Muscle-specific Protein Kinase (MuSK) releases organophosphate aged-acetylcholinesterase (AChE) from C2C12 cells.

Content

Supplementary Table S1. List of antibodies used for Western Blot

Supplementary Figure S1. Full Western Blot images for figure 4B

Supplementary Table S1. List of antibodies used for Western Blot

Name	Blocking solution	Dilution	Manufacturer/ Catalog #
Anti-pMuSK Y755	4% BSA in 0.1% TBSt	1:1000	Invitrogen/ PA5-105382
Anti-MuSK	5% BSA in 0.05% TBSt	1:500	Invitrogen/ MA5-37664
Anti-β-Actin	5% milk in 0.1% TBSt	1:30000	Cell signaling/8457

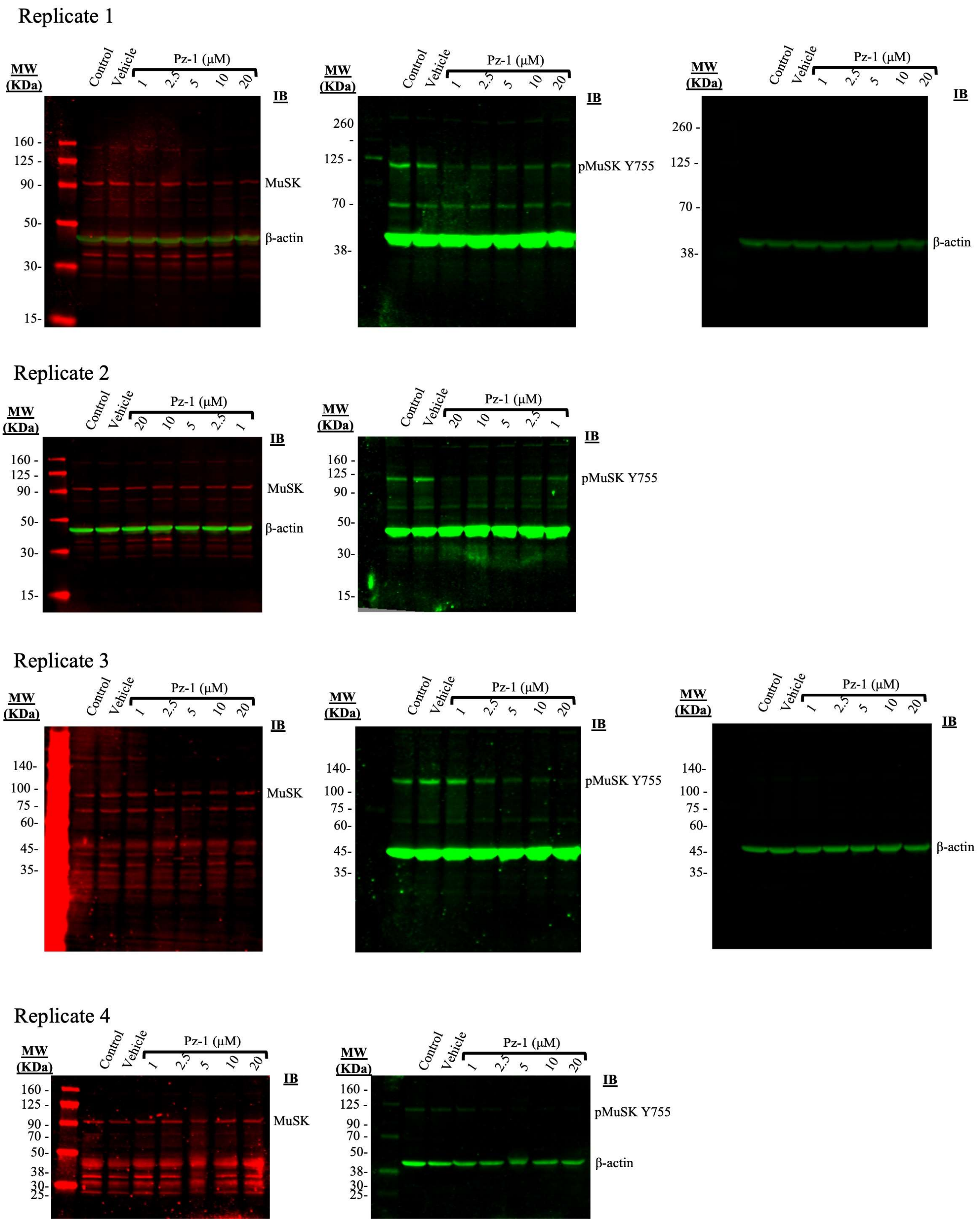

Supplementary Figure S1. Full Western Blot images for figure 4B
